# Systematic review and meta-analysis of cost-effectiveness of minimally invasive versus open pancreatic resections

**DOI:** 10.1007/s00423-023-03017-w

**Published:** 2023-08-12

**Authors:** Suhyun Lee, Chris Varghese, Matthew Fung, Bijendra Patel, Sanjay Pandanaboyana, Bobby V. M. Dasari

**Affiliations:** 1 University of Manchester, Manchester, UK; 2 Department of Surgery, Faculty of Medical and Health Sciences, University of Auckland, Auckland, New Zealand; 3 Institute of Cancer, Barts and the London School of Medicine and Dentistry, London, UK; 4 Queen Mary University of London, London, UK; 5 HPB and Transplant Unit, Freeman Hospital, Newcastle Upon Tyne, UK; 6 Population Health Sciences Institute, Newcastle University, Newcastle Upon Tyne, UK; 7 Department of HBP and Liver Transplant Surgery, Queen Elizabeth Hospital, Edgbaston, Birmingham, B15 2TH UK; 8 Institute of Immunology and Immunotherapy, University of Birmingham, Birmingham, UK

**Keywords:** Cost, Pancreatic resection, Open surgery, Minimally invasive surgery

## Abstract

**Background:**

The systematic review is aimed to evaluate the cost-effectiveness of minimally invasive surgery (MIS) and open distal pancreatectomy and pancreaticoduodenectomy.

**Method:**

The MEDLINE, CENTRAL, EMBASE, Centre for Reviews and Dissemination, and clinical trial registries were systematically searched using the PRISMA framework. Studies of adults aged ≥ 18 year comparing laparoscopic and/or robotic versus open DP and/or PD that reported cost of operation or index admission, and cost-effectiveness outcomes were included. The risk of bias of non-randomised studies was assessed using the Newcastle–Ottawa Scale, while the Cochrane Risk of Bias 2 (RoB2) tool was used for randomised studies. Standardised mean differences (SMDs) with 95% confidence intervals (CI) were calculated for continuous variables.

**Results:**

Twenty-two studies (152,651 patients) were included in the systematic review and 15 studies in the meta-analysis (3 RCTs; 3 case-controlled; 9 retrospective studies). Of these, 1845 patients underwent MIS (1686 laparoscopic and 159 robotic) and 150,806 patients open surgery. The cost of surgical procedure (SMD 0.89; 95% CI 0.35 to 1.43; *I*^2^ = 91%; *P* = 0.001), equipment (SMD 3.73; 95% CI 1.55 to 5.91; *I*^2^ = 98%; *P* = 0.0008), and operating room occupation (SMD 1.17, 95% CI 0.11 to 2.24; *I*^2^ = 95%; *P* = 0.03) was higher with MIS. However, overall index hospitalisation costs trended lower with MIS (SMD − 0.13; 95% CI − 0.35 to 0.06; *I*^2^ = 80%; *P* = 0.17). There was significant heterogeneity among the studies.

**Conclusion:**

Minimally invasive major pancreatic surgery entailed higher intraoperative but similar overall index hospitalisation costs.

**Supplementary Information:**

The online version contains supplementary material available at 10.1007/s00423-023-03017-w.

## Introduction

Surgical resection for pancreatic cancer by means of pancreaticoduodenectomy (PD) and distal pancreatectomy (DP) remains the primary modality of treatment. Traditional resection by open surgery is associated with high perioperative morbidity despite improvements in perioperative care and operative techniques [1, 2]. Minimally invasive approaches (MIS), including robotic and laparoscopic PD and DP, are being increasingly used in pancreatic surgery and are increasingly offered with a hypothesis that it may be associated with lower morbidity, less blood loss, improved surgical margins, and decreased length of hospital stay [3, 4]. The efficacy of both the approaches of MIS (robotic and laparoscopic) for major pancreatic resections is considered similar, and its role in DP is well established [4–6].

The learning curve of MIS for major pancreatoduodenectomies is considered long and remains a major barrier for adaptation among the HPB surgical community. In addition, robotic surgery entails significant perceived upfront and ongoing maintenance costs [7], though there has been some suggestion that is just as cost-effective as laparoscopic pancreatic surgery [8, 9]. It remains unclear how cost-effective MIS is compared to open pancreatic surgery [10–12]. Such economic evaluations are required to guide future policies and guidelines.

This systematic review and meta-analysis therefore aimed to evaluate the cost-effectiveness of minimally invasive versus open surgery for DP or PD.

## Methods

### Literature search

A systematic search of MEDLINE, EMBASE, CENTRAL, Centre for Reviews and Dissemination, and clinical trials registries until April 2020 was reported in accordance with the PRISMA framework [13] (Figure S1). The search strategy included the combination of the terms for ‘pancreatic surgery’, ‘minimally invasive’, ‘open’, ‘cost’, and ‘quality of life’ with Boolean operators OR and AND where appropriate. The full search strategy can be found in the Supplementary Material. No language, publication status, or publication year restrictions were applied. A manual search of references was also conducted to identify any additional relevant literature not captured by the search.

### Inclusion and exclusion criteria

Studies of adults aged ≥ 18 year comparing laparoscopic and/or robotic versus open DP and/or PD that reported cost of operation or index admission and cost-effectiveness outcomes were included. Studies that included subjects aged < 18 years, or other major simultaneous surgeries in addition to DP or PD other than splenectomy, without separate reporting were excluded. Unpublished data, non-peer-reviewed reports, and abstracts were also excluded.

### Data extraction

Two authors independently screened titles and abstracts for full-text inclusion. Article full-texts were also reviewed by two authors independently with conflicts resolved by discussion. Data was extracted onto a prespecified template. Extracted outcomes included costs (cost of surgical procedure, defined as intraoperative costs; surgical instrument costs, operating room occupation costs, index hospitalisation costs, and cost-effectiveness). Quality-adjusted life year (QALY) is a measure of disease burden that incorporates quality and quantity of life.

There are different types of health economic evaluations, including cost-minimisation analysis (CMA), cost-effectiveness analysis (CEA), and cost–benefit analysis. CMA determines an intervention that is least expensive. CEA, including cost-utility analysis (CUA) and cost-consequence analysis, compares interventions that have common outcomes. CUA uses QALY to evaluate the benefit of quality of life and survival time gained against the cost [14]. Incremental cost-effectiveness ratio (ICER) is the difference in total costs (incremental costs) divided by an outcome measure (incremental effect) which provides the extra cost per unit outcome. CMA and ICER were extracted from studies, where reported. Subgroup analysis was performed where combined cost data were reported for laparoscopic and robotic subgroup (‘Lap + Rob’), laparoscopy only (‘Lap-only’), and robotic only (‘Rob-only’) groups.

### Risk of bias

The risk of bias of non-randomised studies was assessed using the Newcastle–Ottawa Scale [15], while the Cochrane Risk of Bias 2 (RoB2) tool was used for randomised studies [16] (Figure S2; Table S1).

### Cost data

Cost data was converted to 2020 US dollars (USD) using a web-based tool (CCEMG-EPPI centre cost converter) [17] when the currency and price-year were different and were available. The costs were converted into the current-year cost of the country using the Gross Domestic Product Deflator Index, followed by conversion into USD for the year 2020. The purchasing power parity for the gross domestic product was used for the conversion rates. The latest reported date of the price year or date of last patient recruitment for surgery was used where price year was not stated. The amortised cost of robotic surgery was not included in meta-analysis. Willingness-to-pay threshold was calculated for ₤20,000 and ₤30,000, as per UK NICE guidelines, adjusted at 2020 price-year. Index hospitalisation cost was considered as the overall payment made towards the index hospital admission.

### Statistical analysis

Review Manager 5.3 [18] (The Nordic Cochrane Centre, Copenhagen, Denmark) was used to perform meta-analyses of cost data. Standardised mean differences (SMDs) with 95% confidence intervals (CI) were calculated for continuous variables. The *I*^2^ statistic was graded as low, moderate, and high heterogeneity when scores were < 30, 30–50, and ≥ 50% respectively [19]. Medians were converted to mean estimates as per Higgins et al. [20]. In cost-meta-analyses, where laparoscopic and robotic groups were reported in separate arms, they were combined into a Lap + Rob group, which was reported as a subgroup analysis as per Higgins et al. [19]. Funnel plots were visually assessed for publication bias (Figure S3).

## Results

The search yielded 1398 articles of which 22 studies were included and 15 studies with cost-data were included in the meta-analysis (Figure S1). Included studies were published between 2008 and 2020; three were randomised controlled trials (RCT) [21–23]; three were case-controlled [10, 24, 25], and the remainder were retrospective studies. Most studies were conducted in the USA [25–36], Italy [10, 37, 38], or Netherlands [21–23]. Overall 152,651 patients were included: 1845 in the MIS group (1686 laparoscopic and 159 robotic) and 150,806 in the open group. DP was performed in 2504 patients: 929 MIS and 1575 open. PD was performed in 150,148 patients: 916 MIS and 149,232 open. Proportion of robotic surgery in MIS cohorts ranged from 10.6 to 71.8%. The study characteristics are summarised in Table 1.

**Table 1:** Characteristics of included studies

Author and year	Country (no. of hospitals)	Design	Quality*	Intervention(s) (*n*)	Control (*n*)
Baker (2015)	USA (1)	Retrospective cohort	9	RPD (22)	OPD (49)
Braga (2015)	Italy (1)	Case-controlled study	9	LDP (100)	ODP (100)
de Rooij (2019)	Netherlands (14)	Randomised controlled trial	-	MIDP (47 – LDP 42, RDP 5)	ODP (55)
Eom (2008)	South Korea (2)	Case-controlled study	8	LDP (31)	ODP (62)
Fisher (2019)	USA (National study)	Retrospective cohort	8	1) LDP (146) 2) RDP (53)	ODP (693)
Fox (2012)	Canada (1)	Retrospective cohort	7	LDP (42)	ODP (76)
Gerber (2017)	USA (1)	Retrospective cohort	8	LPD (52)	OPD (*n* = 50)
Langan (2014)	USA (1)	Case-controlled study	8	LDP (41)	ODP (40)
Liang (2015)	Canada (1)	Retrospective cohort	6	LPD (15)	OPD (29)
Limongelli (2012)	Italy (1)	Retrospective cohort	9	LDP (16)	ODP (29)
Mesleh (2013)	USA (1)	Retrospective cohort	8	LPD (59)**	OPD (43)**
Ricci (2015)	Italy (1)	Retrospective cohort	8	LDP (41)	ODP (40)
Rodriguez (2018)	France (2)	Retrospective cohort	7	1) LDP (25) 2) RDP (21)	ODP (43)
Rutz (2014)	USA (1)	Retrospective cohort	9	LDP (70)	ODP (45)
Stewart (2020)	USA (1)	Retrospective cohort	7	MIPD 39 (LDP 11, RDP 28)	ODP (41)
Torphy (2019)	USA (1)	Retrospective cohort	7	LDP (26)	ODP (77)
Tran (2016)	USA (National study)	Retrospective cohort	8	LPD (681)	OPD (14,893)
van Hilst (2019)	Netherlands (14)	Randomised controlled trial	-	LPD (50)	OPD (49)
van Hilst (2019a)	Netherlands (14)	Randomised controlled trial	-	LDP (43)	ODP (56)
Waters (2010)	USA (1)	Retrospective cohort	8	1) LDP (18) 2) RDP (17)	ODP (22)
Xourafas (2015)	USA (1)	Retrospective cohort	9	LDP (56)	ODP (67)
Xourafas (2019)	USA (1)	Retrospective cohort	9	MIDP (97 – LDP 67, RDP 30)	ODP (128)

*LDP* laparoscopic distal pancreatectomy, *LPD* laparoscopic pancreaticoduodenectomy, *MIDP* minimally invasive distal pancreatectomy, *MIPD* minimally invasive pancreaticoduodenectomy, *ODP* open distal pancreatectomy, *OPD* open pancreaticoduodenectomy, *RDP* robotic pancreaticoduodenectomy

^*^Newcastle–Ottawa Quality Assessment Scale; **excludes total pancreatectomy (*n* = 16 for LPD and *n* = 5 for OPD)

### Clinical characteristics

Patient age, gender, tumour characteristics, rates of previous abdominal surgery, and American Society of Anaesthesiologists (ASA) classification > 3 were comparable between MIS and open groups (Table 2).

**Table 2:** Clinical characteristics of patients included undergoing minimally invasive or open major pancreatic surgery

Author year	Male (%)			Mean age (SD)			Mean BMI (SD)			Prev. abdominal surgery (%)			ASA > III			Diagnosis/tumour characteristics		
	MI	Open	*P*	MI	Open	*P*	MI	Open	*P*	MI	Open	*P*	MI	Open	*P*	MI	Open	*P*
Baker (2015)	5.10%	62.90%	NS	63 (38–82)*	63 (26–86)*	NS	25.5 (18.2–35.1)*	26.7 (16.2–38.2)*	NS	45.50%	49%	NS	68.10%	81.60%	NS	> 65% pancreatic adenoCa	> 65% pancreatic adenoCa	NS
Braga (2015)	44%	44%	NS	61.4 (13.5)	61.0 (13.8)	NS	NR	NR	NS	-	-	-	12%	17%	NS	30% pancreatic adenoid; 28% endocrine tumour; 14% mucinous cystadenoma; 14% IPMN	34% pancreatic adenoid; 29% endocrine tumour; 8% large serous cystadenoma; 7% IPMN	NS
de Rooij (2019)	57%	28%	NS	61 (13)	63 (12)	NS	27 (6)	26 (4)	NS	41%	48%	NS	14%	18%	NS	31% NET; 25% pancreatic ductal adenoCa; 22% cystic tumour	39% NET; 18% pancreatic ductal adenoCa; 36% cystic tumour	NS
Eom (2008)	-	-	-	46.7 (16.7)	47.5 (14.9)	NS	22.2 (2.2)	23.0 (3.4)	-	-	-	-	-	-	-	26% mucinous cystic neoplasm; 26% solid pseudopapillary neoplasm	26% mucinous cystic neoplasm; 24% solid pseudopapillary neoplasm; 19% intraductal papillary mucinous neoplasm	NS
Fisher (2019)	45%	46%	NS	58 (14)	61 (15)	0.04	-	-	-	-	-	-	-	-	-	NA	NA	NA
Fox (2012)	31%	51.30%	0.036	55.3 (16.4)	58.4 (14.4)	NS	-	-	-	54.80%	42.10%	NS	-	-	-	33.3% NET; 14.3% IPMT; 14.3% mucinous tumours; 11.9% SPEN; 11.9% cyst	23.7% NET; 21.1% solid mass of uncertain behavious; 19.7% cyst; 25% malignant mass	< 0.001
Gerber (2017)	-	-	-	-	-	-	-	-	-	-	-	-	-	-	-	Periampullary adenoCa	Periampullary adenoCa	NA
Langan (2014)	39%	48%	-	64 (45–84)*	65 (34–85)*	-	27.78*	22.58*	-	-	-	-	-	-	-	64% malignant	57% malignant	Not reported
Limongelli (2012)	60%	88.50%	NS	62.1 (6.9)	64.1 (5.8)	NS	26.4 (2.5)	27.1 (2.1)	NS	-	-	-	-	-	NS	Mucinous cystadenoma/adenoCa; serous cystadenoma/adenoCa; IPMN; NET	Mucinous cystadenoma/adenoCa; serous cystadenoma/adenoCa; IPMN; NET; epithelial cyst; ductal adenoCa	NS
Liang (2015)	27%	55%	NS	67 (26–82)*	65 (20–77)*	NS	25 (21–35)*	28 (22–45)*	0.003	-	-	-	93%	100%	NS	60% malignant	79% malignant	NS
Mesleh (2013)	57%	48%	NS	-	-	NS	-	-	NS	-	-	-	80%	92%	NS	77% periampullary adenoCa	79% periampullary adenoCa	NS
Ricci (2015)	34%	53%	NS	58	67	NS	25.3	26.6	NS	56.10%	70%	NS	-	-	-	56% benign; 44% low-grade malignant disease	45% benign; 55% low-grade malignant disease	NS
Rodriguez (2018)	39%	51.20%	NS	R: 54 (27–79)*	65 (38–86)	0.005	R: 25(18–33)*	24.7 (17–34)	NS	70%	72.10%	NS	15%	18.60%	NS	R: 61.9% malignant (38.1% NET); 38.1% benign (23.8% mucinous cystadenoma)	79.1% malignant (51.2% adenoCa); 20.9% benign (9.3% IPMN; 7% mucinous cystadenoma)	NS
				L: 62.5 (27–83)*			L: 27.3 (20–41)*									L: 68% malignant (32% adenoCa; 36% NET); 32% benign (16% IPMN; 12% pancreatitis)		
Rutz (2014)	34%	47%	NS	58.6 (13.5)	56.3 (16.1)	NS	-	-	-	-	-	-	-	-	NS	31% serous/mucinous cystadenoma; 26% NET; 16% adenoCa	24% NET; 18% adenoCa; 18% serous/mucinous cystadenoma; 18% other	NS
Stewart (2020)	32%	39%	0.15	-	69 (39–84)*	NS	28.4 ((5.2)	26.4 (5.7)	NS	-	-	-	-	-	-	52% malignant	83% malignant	0.04
Torphy (2019)	40%	50%	NS	-	-	NS	-	-	-	-	-	-	-	-	-	65% cancer; 35% benign	76% cancer; 24% benign	NS
Tran (2016)	55%	52%	NS	67 (58–73)	65 (56–73)**	0.001	-	-	-	-	-	-	-	-	-	NA	NA	NA
van Hilst (2019)	40%,	51%	-	67	66	-	25 (3)	26 (4)	-	32%,	25%	-	26%	33%	-	28% pancreatic ductal adenoCa; 24% ampullary tumour; 16% IPMN	31% pancreatic ductal adenoCa; 18% IPMN; 16% cholangiocarcinoma; 12% ampullary tumour	NS
				(59–76)**	(61–73)**													
van Hilst (2019a)	60%	48%	NS	61 (13)	63 (12)	NS	27 (6)	26 (4)	NS	42%	48%	NS	15%	18%	NS	NA	NA	NA
Waters (2010)	50%	45%	NS	59	59	NS	-	-	-	-	-	-	-	-	NS	R: 35% IPMN; 29% NET; 18% MCN L: 28% NET; 17% MCN	50% AdenoCa; 18% NET; 18% IPMN	NS
Xourafas (2015)	56%	49%	NS	61 (20–95)*	62 (34–92)*	NS	27.8*	28.4*	NS	-	-	-	-	-	-	88% nonfunctioning; 11% insulinoma	72% nonfunctioning; 21% insulinoma	0.01 for non-functioning; NS for insulinoma
Xourafas (2019)	40%	50%	NS	63 (49–69)**	60 (50–68)**	NS	28.7 (26–33)**	27.6 (24–31)**	NS	-	-	-	57%	70%	NS	43% malignant (10% pancreatic adenoCa)	55% malignant (18% pancreatic adenoCa)	NS

*ASA* American Society of Anaesthesiologists status, *BMI* body mass index, *MI* minimally invasive, *SD* standard deviation, *NS* not significant, *NR* not reported

^*^Median (range); **median (IQR)

### Economic evaluation

Twenty studies [8, 10, 21–24, 26–28, 30, 32–41] compared cost via CMA between MIS and open major pancreatic surgery; this is summarised in Table 3. All economic evaluations were trial based; none used model-based approaches.

**Table 3:** Summary of economic evaluation in each study

Author year	Intention-to-treat	Economic analysis	Perspective	Currency; Price-year;	Direct/indirect Costs; Source; Unit cost	Effectiveness/ICER
Baker 2015	Yes	CMA	NR	USD;	Direct cost;	-
				Price-year not stated;	Cost source not stated;	
					Unit cost not stated	
Braga 2015	Yes	CMA	NR	Euros;	Direct costs	-
				Price year not stated;	Cost source not stated;	
					Unit cost not stated	
de Rooij 2019	Yes	CMA	Healthcare	USD;	Direct costs	-
				2018;	Cost source not stated;	
					Unit cost not stated	
Eom 2008	Not stated	CMA	NR	USD;	Direct cost	-
				Price year not stated;	Cost source not stated;	
					Unit cost not stated	
Fisher 2019	As-treated	CMA	Healthcare Payer’s	USD;	Direct cost	-
				2014;	Cost source stated;	
					Unit cost not stated	
Fox 2012	Yes	CMA	NR	CAD;	Direct costs	-
				2010;	Cost source stated for some.	
					Unit cost not stated	
Gerber 2017	Yes	CMA	NR	USD;	Direct cost	-
				Price-year not stated;	Cost source: stated	
					Unit cost not stated;	
Liang 2015	Not stated	CMA	NR	CAD;	Direct cost;	-
				Price-year not stated	Cost source not stated;	
					Unit cost not stated	
Limongelli 2012	Yes	CMA	NR	Euros;	Direct cost;	-
				price-year not stated	Cost source not stated;	
					Unit cost not stated	
Mesleh 2013	Yes	CMA	NR	“units” by a set conversion factor;	Direct cost;	-
				Price-year not stated;	Cost source not stated;	
					Unit cost not stated	
Ricci 2015	Yes	CMA, CUA	NR	Euro;	Direct cost;	QALY
				Price-year not stated;	Cost source not stated;	ICER
					Unit cost not stated	
Rodriguez 2018	Yes	CMA	NR	Euro;	Direct cost;	-
				Price-year not stated;	Cost source: stated	
					Unit cost: stated for one category only.	
Rutz 2014	Yes	CMA	NR	USD;	Direct cost;	-
				Price-year not stated;	Cost source: stated for some;	
					Unit cost not stated	
Stewart 2020	Converted cases as separate category	CMA	NR	USD;	Direct cost	-
				2016;	Cost source: stated	
					Unit cost: not stated	
Tran 2016	Not stated	CMA	NR	USD;	Direct cost	-
				Price-year adjusted from 2000 to 2010;	Cost source: stated	
					Unit cost: not stated	
van Hilst 2019	Yes	CMA	Healthcare	USD;	Direct cost	-
				Price-year not stated;	Cost source: not stated	
					Unit cost: not stated	
van Hilst 2019a	Yes	CMA, CEA, CUA	Healthcare	Euro;	Direct cost;	-*
				2016;	Cost source: stated	
					Unit cost: stated	
Waters 2010	Yes	CMA	NR	USD;	Direct cost and indirect cost of robotic purchase and maintenance.	-
				price-year not stated;	Cost source: not stated	
					Unit cost not stated	
Xourafas 2015	Yes	CMA	NR	USD;	Direct cost	-
				Price-year not stated;	Cost source: stated	
					Unit cost: not stated	
Xourafas 2019^(127^^**)**^	Yes	CMA	NR	USD;	Direct cost and indirect cost of robotic maintenance.	-
				Price-year not stated;	Cost source: stated	
					Unit cost: not stated	

*USD* US dollar, *CE* cost-effectiveness analysis, *CMA* cost-minimisation analysis, *CUA* cost-utility analysis

^*^Time to functional recovery /QALY/ICER measured against total cost until after discharge (i.e. outpatients and emergency room)

#### Cost of surgical procedure

**Fig. 1:**
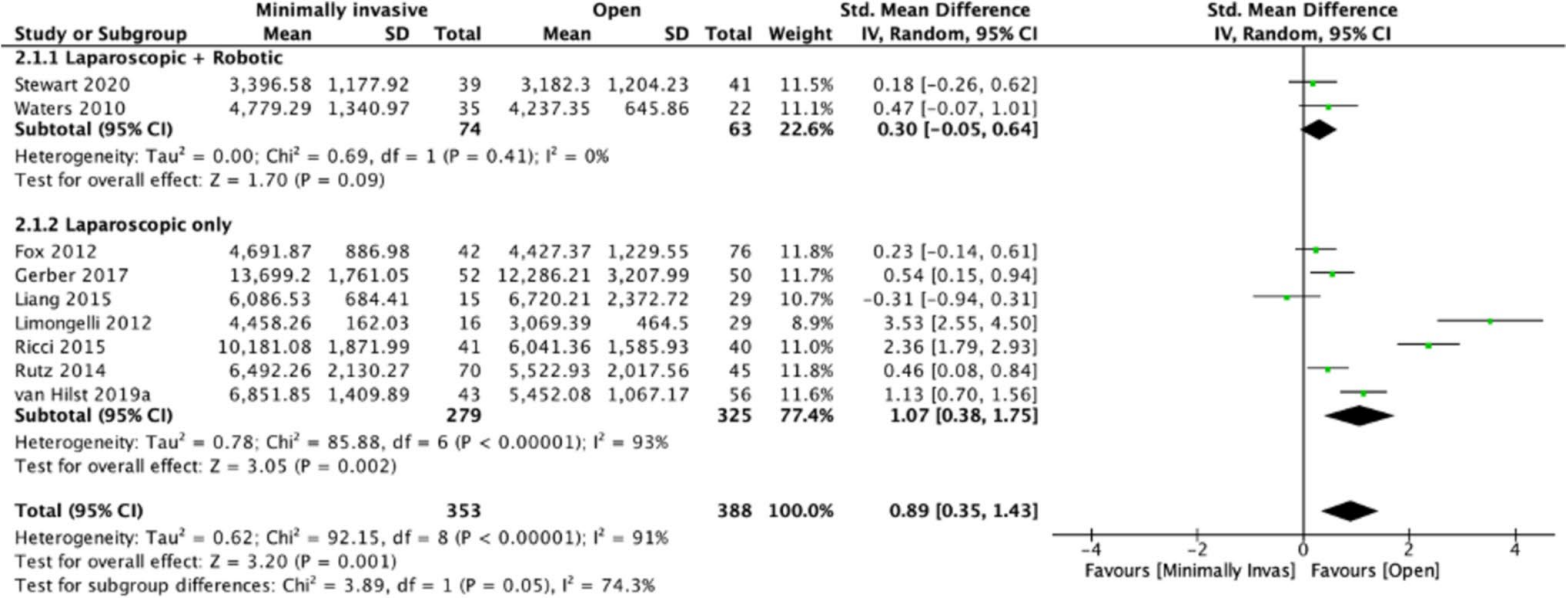
Overall cost of surgery open vs. MIS surgery

Fifteen studies [21–24, 27, 28, 30, 32–34, 37–41] were included in a meta-analysis of cost; nine assessed the cost of the surgical procedure. Mean cost of MIS was $6737.436 (range 3396.59 to 13,699.2) and open surgery was $5659.911 (3069.39 to 12,286.21). Operative cost significantly favoured open surgery (SMD 0.89, 95% CI 0.35 to 1.43; *I*^2^ = 91%; *P* = 0.001); however, there was high heterogeneity (Fig. 1). Subgroup analysis of Lap + Rob (*n* = 2; 48.6 and 71.8% robotics in each study respectively) showed that open surgery was favoured but was not significant (SMD 0.30, 95% CI − 0.05 to 0.64; *I*^2^ = 0%; *p* = 0.09). The Lap-only (*n* = 7) subgroup analysis showed open surgery was favourable (SMD 1.07, 95% CI 0.38 to 1.75; *I*^2^ = 91%; *p* = 0.002), but high heterogeneity persisted (Fig. 1).

Five studies [8, 10, 26, 35, 36] were not included in meta-analysis of operative cost; Rodriguez et al. did not report separated costs of the surgical procedure alone [8]. Xourafasb et al. found open surgery to be 20% more expensive than laparoscopy (*P* = 0.091) [35]. In the remaining three studies, MIS had higher operative cost; Lap + Rob (MIS was 16% more expensive; *P* < 0.001; [36]), Lap-only (mean difference + $1451; [10]), or Rob-only (median difference + $20,543; *P* < 0.001; [26]).

#### Cost of surgical instruments

**Fig. 2:**
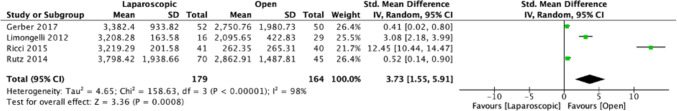
Comparison of instrument costs

Four studies [28, 30, 37–39] assessed the cost of surgical instruments; mean equipment cost of laparoscopic surgery was $3402.10 (range 3208.28 to 3798.42) and open surgery was $1992.918 (262.35 to 2862.91). Material cost significantly favoured open surgery (SMD 3.73, 95% CI 1.55 to 5.91; *I*^2^ = 98%; *P* = 0.0008); however, there was high heterogeneity (Fig. 2). Rodriguez et al. report highest surgical equipment costs with robotic surgery (median $2871 (range 2507–3724) vs $48 and $38 for laparoscopic and open respectively) [8].

#### Cost of operating room

**Fig. 3:**
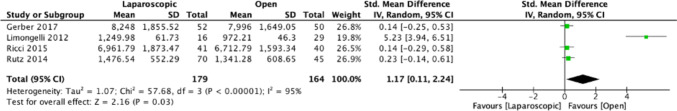
Comparison of operating room costs

Four studies [28, 30, 37, 38] assessed costs of operating room (OR) occupation; mean OR occupation cost of laparoscopic surgery was $4484.13 (range 1476.54 to 8248.21) and open surgery was $4255.57 (972.21 to 7996). OR occupation cost also significantly favoured open surgery (SMD 1.17, 95% CI 0.11 to 2.24; *I*^2^ = 95%; *P* = 0.03); however, there was high heterogeneity (Fig. 3).

#### Cost of index hospitalisation

**Fig. 4:**
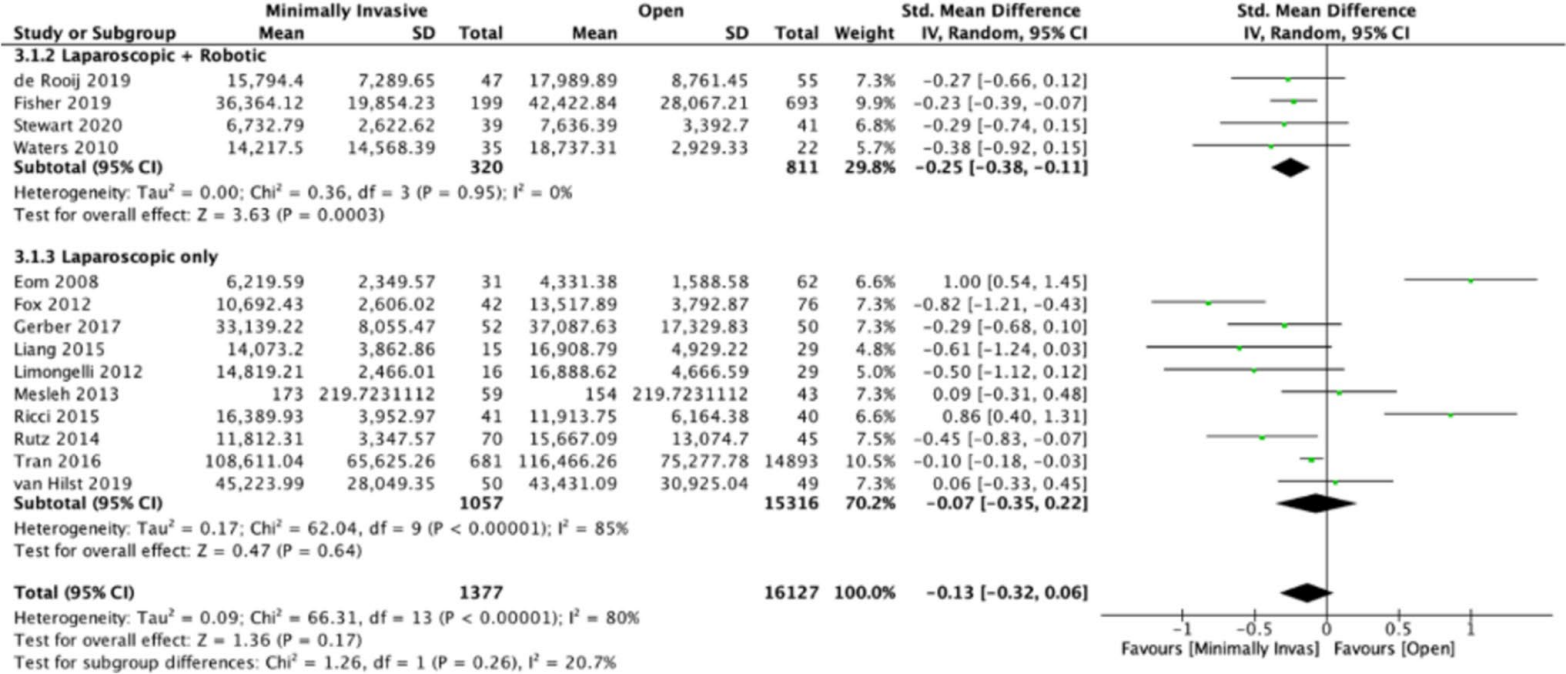
Comparison of index hospitalisation costs

Fourteen studies [21, 22, 24, 27–30, 32–34, 37–40] assessed the cost of index hospitalisation; mean cost of MIS was $25,699.21 (range 6219.59 to 108611), and open surgery was $27,922.99 (4331.38 to 11,6466.3). MIS was favoured on meta-analysis, but the benefit was not statistically significant (SMD − 0.13, 95% CI − 0.32 to 0.06; *I*^2^ = 80%; *P* = 0.17). There was high heterogeneity (Fig. 4). Lap + Rob subgroup analysis (*n* = 4) favoured MIS (SMD − 0.25, 95% CI − 0.38 to − 0.11; *I*^2^ = 0%; *P* = 0.003). Results also favoured MIS in the Lap-only subgroup, but this did not reach statistical significance and was associated with high heterogeneity (SMD − 0.07, 95% CI − 0.35 to 0.22; *I*^2^ = 85%; *P* = 0.64).

Five studies were not included in the meta-analyses. Amongst these, one demonstrated lowest cost with robotic surgery followed by laparoscopic and highest with open surgery (*P* = 0.02) [8]. This was found to be the case even after adjusting for cost of robotic maintenance [36]. The remaining three studies found no significant differences in costs of index hospitalisation [10, 26, 42] (Table S3).

#### Cost-effectiveness

Ricci et al. conducted cost-utility analysis in addition to CMA using EQ-5D derived QALY as a measure of effectiveness [37]. They showed a mean difference in QALY was 0.2 ± 0.08 (*P* = 0.005), and total index hospitalisation cost was €1379 ± 919; *P* < 0.001), suggesting the greater cost of laparoscopic surgery was balanced by the improved benefit in QOL [37]. The probability for laparoscopy being more cost-effective than open was higher at a willingness to pay above €5400 per QALY. The ICER was €5622 (₤5808 for 2020 price-year) per QALY gained. Van Hilst et al. were excluded from cost-effectiveness analysis as the mean cost difference was derived from total cost including time after discharge [23].

#### Laparoscopic versus robotic techniques

Rodriquez et al. reports higher costs associated with robotic surgery for cost of surgery, OR occupation, surgical instruments, but not overall index hospitalisation [8]. Two studies also showed higher costs of OR occupation for MIS (robotic > laparoscopic > open), with laparoscopic surgery costing $338 more per patient [8, 10]. This was similar amongst non-meta-analysed studies that report greater instrument costs for MIS (robotic > laparoscopic [8]) compared to open by between 65 and 93% and greater costs per person [10, 36, 42] (Table S2).

### Risk of bias

Observational studies overall had a low risk of bias, as most scored between 6 and 8 on the Newcastle–Ottawa quality assessment scale (Table 1). The included RCTs were overall associated with a high risk of bias largely due to outcome reporting [21–23]. A summary of the risk of bias assessment can be found in Table S1 and Figure S2. Index hospitalisation was best reported in the lap subgroup and had enough studies to assess publication bias; see Figure S3.

## Discussion

This systematic review and meta-analysis evaluated the available evidence on the costs and cost-effectiveness of MIS and open major pancreatic surgery. Cost of surgical procedure, cost of surgical instruments, and operating room costs significantly favoured open surgery; however, the overall cost of index hospitalisation was comparable between open surgery and MIS. The reasons for reduced hospitalisation are not detailed and could be because of advantages in reduced length of stay or the operator bias.

Cost of surgical instruments was significantly more expensive for MIS than open surgery despite the exclusion of the cost of purchase and maintenance of robotic equipment. This is similar in other areas with established robotic surgical practice such as prostatectomy where costs of surgical supplies and OR occupation are higher with MIS compared to open prostatectomy, despite excluding amortised cost and reduced operative time [43, 44]. The requirement for additional equipment such as laparoscopic and robotic electrosurgical instruments and needle drivers may be a key contributor to these raised costs [45, 46]. Childers and Maggard-Gibbons found that instrument and accessory costs of robotics across gynaecological, urological, and general surgeries were around $1866 per procedure [47], whereas equipment for non-robotic surgery is relatively inexpensive. Our findings show that robotic surgery had the highest costs, followed by laparoscopic then open, confirming the results of a recent meta-analysis comparing robotic and laparoscopic DP [48, 49].

Despite significantly greater costs of MIS for DP and PD, overall cost of index hospitilisation was comparable in the laparoscopic and robotic subgroup. Higher operative costs of MIS may be compensated by lower costs outside of the OR. This is likely related to decreased length of hospital stay and reduced recovery times [50, 51]. Previous meta-analysis found a laparoscopic approach to DP reduced length of stay by 3.8 days (*P* < 0.01) compared to open [52]. Abu Hilal et al. [53] showed similar findings: lower postoperative costs with laparoscopic compared to open DP (− £5547, *P* = 0.006) resulting in an overall reduction in hospitalisation cost of £4737 (*P* = 0.197). Similarly, Fingerhut et al. compared laparoscopic vs open DP [54] and showed higher operative but lower postoperative costs resulting in cost-equipoise between both approaches.

The current study found that MIS is associated with an incremental cost of €5622 per QALY gained. This meets the threshold adapted by the NHS in the UK of £20,000 and £30,000 per QALY for recommending treatments [55]. MIS had a 100% probability of being cost-effective when the threshold value for an additional gain of QALY was £20,000. As experience with MIS increases and the availability of robotic surgery increases, the cost-effectiveness may further improve and may justify its use in the healthcare systems that are interested in delivering equitable and sustainable value-based healthcare.

There are several limitations of the included studies in this review. Most studies were case-controlled and three were RCTs. There was also a high level of heterogeneity between studies, potentially due to the studies being conducted across 6 countries where costs vary between hospitals and healthcare systems. Because of this, the applicability of the average cost of MIS and open surgery may have limited transferability into individual healthcare settings [56]. Reporting the resection of proximal and distal pancreatic resections with different operative complexity and outcomes also adds to the heterogeneity. To minimise bias due to this heterogeneity, we decided to use SMD to measure effect sizes. Two of the studies had high heterogeneity; a sensitivity analysis performed has not changed the overall results of meta-analyses [37, 38]. Direct cost assessment, as done in this review, does not account for indirect benefits such as earlier return to normal activities including and loss of productivity [57]. This study also does not account for the training costs associated with the significant learning curve associated with laparoscopic and robotic surgery [58]. The small sample size of most studies also necessitates further research to detect differences between MIS and open surgery. Also, the overall number of robotic procedures is small and will need future larger studies. The similar overall cost of index hospitalisation suggests minimally invasive pancreatic surgery is no worse than open approaches. However, only one included study analysed the ICER of laparoscopic DP, and further studies are required to evaluate the cost-effectiveness of the MIS approach to pancreatic surgery. Given the smaller volumes in the minimally invasive cohort, the authors discussed and opted to keep the laparoscopic and robotic options together. There is a need to perform cost-analysis of robotic surgery separately as the data evolves and the procedure gets established. Publication bias was evident in the laparoscopic-only subgroup analysis of index hospitalisation costs. Hence, the results from this analyses have to be interpreted with much caution. The authors believe that the limitations of this review would highlight the drawbacks and help in the development of appropriate methodology for future studies.

In conclusion, this systematic review and meta-analysis found that MIS approaches are associated with higher procedural costs but may have similar overall index hospitalisation costs compared to open pancreatic surgery.

### Supplementary Information

Below is the link to the electronic supplementary material.

Supplementary file1 (DOCX 229 KB)
