# Supplementary material for: Systematic review and meta-analysis of cost-effectiveness of minimally invasive versus open pancreatic resections
Source: Langenbecks Arch Surg. 2023 Aug 12;408(1):306. doi: 10.1007/s00423-023-03017-w (PMC10423165; doi:10.1007/s00423-023-03017-w)
Supplement: Supplementary file 1 — Supplementary file1 (DOCX 229 KB) [file 423_2023_3017_MOESM1_ESM.docx]

**Systematic review and meta-analysis of cost-effectiveness and quality of life following minimally invasive *versus* open major pancreatic resections – Supplementary Material**

Suhyun Lee, Chris Varghese, Alexander Oh, Matthew Fung, Bijen Patel, Sanjay Pandanaboyana, Bobby VM Dasari

**Search Strategy**

1. **Ovid MEDLINE(R) and Epub Ahead of Print, In-Process & Other Non-Indexed Citations and Daily <1946 to March 09, 2020>**

1 Pancreatectomy/

2 pancreatectom$.mp.

3 exp pancreaticoduodenectomy/

4 pancreaticoduodenectom$.mp.

5 pancreatoduodenectom$.mp.

6 duodenopancreatectom$.mp.

7 whipple.mp.

8 (pylorus adj3 preserv$).mp.

9 or/1-8

10 open.mp.

11 laparoscop$.mp.

12 exp Laparoscopy/

13 Robotic Surgical Procedures/

14 (robotic$ or robot or robots).mp.

15 Surgery, Computer-Assisted/

16 (computer assisted or remote$ operat$).mp.

17 da vinci.mp.

18 robotic surgical system$.mp.

19 exp Minimally Invasive Surgical Procedures/

20 (Minimally Invasive or minimal access).mp.

21 or/10-20

22 9 and 21

23 "Quality of Life"/

24 quality-adjusted life years/

25 patient reported outcome measures/

26 exp Health Status/

27 exp Self Concept/

28 "Activities of Daily Living"/

29 Mental Health/ (36683)

30 exp Mental Disorders/

31 Social Adjustment/

32 Pain Measurement/

33 ("Quality of Life" or quality-adjusted life year$ or patient reported outcome$ or Health Status or Self Concept or "Activities of Daily Living" or Mental Health or social adjustment$ or social adaptation).mp.

34 (pain adj3 measur$).mp.

35 (qol or life quality or daily living activit$ or quality adjusted survival or hrql or hrqol or qaly or prom or proms).mp.

36 (health adj3 level).mp.

37 (wellness or wellbeing or well-being).mp.

38 (functional abilit$ or good health or healthiness).mp.

39 (physical$ adj3 limit$).mp.

40 (functional assessment$ or psychiatric status).mp.

41 (fact questionnaire or fact survey or Mental health or rand-36 or sf-36 or short form-36 or facit or toi or eq-5d or eortc or qlq-c30 or Qlq-pan26 or pNET or QLQ-GINET21 or QlQ-C15-PAL or Fact-g or Fact-hep).mp.

42 (mental disorder$ or mental health or depression or depressive or anxiety or mood disorder$).mp.

43 (psychological or psychiatric).mp.

44 Cost-Benefit Analysis/

45 (cost effective$ or cost-effective$ or cost benefit$ or cost-benefit$ or economic$ or less costly or more costly).mp.

46 or/23-45

47 22 and 46

1. **Embase <1974 to 2020 March 10>**

1 exp pancreatectomy/

2 pancreatectom$.mp.

3 exp pancreaticoduodenectomy/

4 pancreaticoduodenectom$.mp.

5 pancreatoduodenectom$.mp.

6 duodenopancreatectom$.mp.

7 whipple.mp.

8 (pylorus adj3 preserv$).mp.

9 or/1-8

10 open.mp.

11 laparoscop$.mp.

12 exp Laparoscopy/

13 robotic surgical procedure/

14 (robotic$ or robot or robots).mp.

15 computer assisted surgery/

16 (computer assisted or remote$ operat$).mp.

17 da vinci.mp.

18 robotic surgical system/

19 exp Minimally Invasive Surgical Procedures/

20 (Minimally Invasive or minimal access).mp.

21 or/10-20

22 9 and 21

23 "quality of life"/

24 quality adjusted life year/

25 patient-reported outcome/

26 exp Health Status/

27 exp Self Concept/

28 daily life activity/

29 exp mental health/

30 exp mental disease/

31 exp social adaptation/

32 exp pain measurement/

33 ("Quality of Life" or quality-adjusted life year$ or patient reported outcome$ or Health Status or Self Concept or "Activities of Daily Living" or Mental Health or social adjustment$ or social adaptation).mp.

34 (pain adj3 measur$).mp.

35 (qol or life quality or daily living activit$ or quality adjusted survival or hrql or hrqol or qaly or prom or proms).mp.

36 (health adj3 level).mp

37 (wellness or wellbeing or well-being).mp.

38 (functional abilit$ or good health or healthiness).mp.

39 (physical$ adj3 limit$).mp.

40 (functional assessment$ or psychiatric status).mp.

41 (fact questionnaire or fact survey or Mental health or rand-36 or sf-36 or short form-36 or facit or toi or eq-5d or eortc or qlq-c30 or Qlq-pan26 or pNET or QLQ-GINET21 or QlQ-C15-PAL or Fact-g or Fact-hep).mp.

42 (mental disorder$ or mental health or depression or depressive or anxiety or mood disorder$).mp.

43 (psychological or psychiatric).mp.

44 economic evaluation/ or "cost benefit analysis"/ or "cost effectiveness analysis"/

45 (cost effective$ or cost-effective$ or cost benefit$ or cost-benefit$ or economic$ or less costly or more costly).mp.

46 or/23-45

47 22 and 46

1. **Cochrane library Database**

#1 MeSH descriptor: [Pancreatectomy] this term only

#2 pancreatectom* 650

#3 MeSH descriptor: [Pancreaticoduodenectomy] explode all trees

#4 pancreaticoduodenectom*

#5 pancreatoduodenectom*

#6 duodenopancreatectom*

#7 whipple

#8 pylorus near/3 preserv*

#9 #1 or #2 or #3 or #4 or #5 or #6 or #7 or #8

#10 open

#11 laparoscop*

#12 MeSH descriptor: [Laparoscopy] explode all trees

#13 MeSH descriptor: [Robotic Surgical Procedures] this term only

#14 robotic* or robot or robots

#15 MeSH descriptor: [Surgery, Computer-Assisted] this term only

#16 "computer assisted" or "remote* operat*"

#17 da vinci

#18 robotic surgical system*

#19 MeSH descriptor: [Minimally Invasive Surgical Procedures] explode all trees

#20 "Minimally Invasive" or "minimal access"

#21 #10 or #11 or #12 or #13 or #14 or #15 or #16 or #17 or #18 or #19 or #20

#22 #9 and #21

#23 MeSH descriptor: [Quality of Life] this term only

#24 MeSH descriptor: [Quality-Adjusted Life Years] this term only

#25 MeSH descriptor: [Patient Reported Outcome Measures] this term only

#26 MeSH descriptor: [Health Status] explode all trees

#27 MeSH descriptor: [Self Concept] explode all trees

#28 MeSH descriptor: [Activities of Daily Living] this term only

#29 MeSH descriptor: [Mental Health] this term only

#30 MeSH descriptor: [Mental Disorders] explode all trees

#31 MeSH descriptor: [Social Adjustment] this term only

#32 MeSH descriptor: [Pain Measurement] this term only

#33 "Quality of Life" or "quality-adjusted life year*" or "patient reported outcome*" or "Health Status" or "Self Concept" or "Activities of Daily Living" or "Mental Health" or "social adjustment*" or "social adaptation"

#34 pain near/3 measur*

#35 qol or "life quality" or "daily living activit*" or "quality adjusted survival" or hrql or hrqol or qaly or prom or proms

#36 health near/3 level

#37 wellness or wellbeing or well-being

#38 "functional abilit*" or "good health" or healthiness

#39 physical* near/3 limit*

#40 "functional assessment*" or "psychiatric status"

#41 "fact questionnaire" or "fact survey" or "rand-36" or "sf-36" or "short form-36" or facit or toi or "eq-5d" or eortc or "qlq-c30" or "Qlq-pan26" or pNET or "QLQ-GINET21" or "QlQ-C15-PAL" or "Fact-g" or "Fact-hep"

#42 "mental disorder*" or "mental health" or depression or depressive or anxiety or "mood disorder*"

#43 psychological or psychiatric

#44 MeSH descriptor: [Cost-Benefit Analysis] this term only

#45 "cost effective*" or "cost-effective*" or "cost benefit*" or "cost-benefit*" or economic* or "less costly" or "more costly"

#46 #23 or #24 or #25 or #26 or #27 or #28 or #29 or #30 or #31 or #32 or #33 or #34 or #35 or #36 or #37 or #38 or #39 or #40 or #41 or #42 or #43 or #44 or #45

#47 #22 and #46

1. **ICTRP Database**

(pancreatectomy OR pancreatectomies OR pancreaticoduodenectomy OR pancreaticoduodenectomies OR pancreatoduodenectomy OR pancreatoduodenectomies OR duodenopancreatectomy or duodenopancreatectomies OR whipple) AND (quality of life OR health status OR well-being OR wellbeing OR psychiatric OR mental OR cost)

1. **York CRD Database**

Results for: (pancreatectomy OR pancreatectomies OR pancreaticoduodenectomy OR pancreaticoduodenectomies OR pancreatoduodenectomy OR pancreatoduodenectomies OR duodenopancreatectomy or duodenopancreatectomies OR whipple) AND (quality of life OR health status OR well-being OR wellbeing OR psychiatric OR mental OR cost)

1. **ClinicalTrials.gov**

Results for: (pancreatectomy OR pancreatectomies OR pancreaticoduodenectomy OR pancreaticoduodenectomies OR pancreatoduodenectomy OR pancreatoduodenectomies OR duodenopancreatectomy or duodenopancreatectomies OR whipple) AND (quality of life OR health status OR well-being OR wellbeing OR psychiatric OR mental OR cost)

**Figure S1: PRISMA flow diagram of study selection**

**
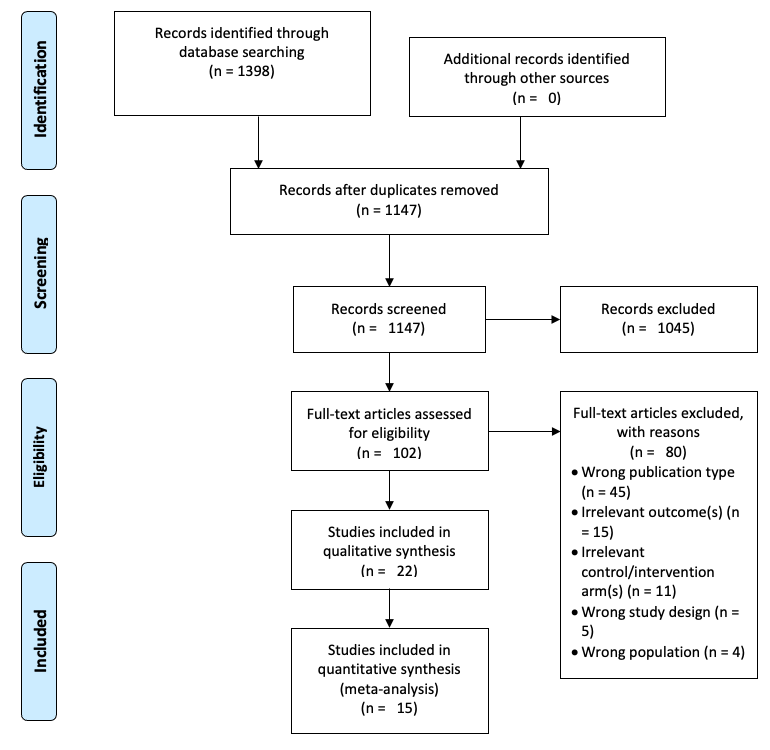
**

**Figure S2. Cochrane risk-of-bias II assessment of RCTs**

**
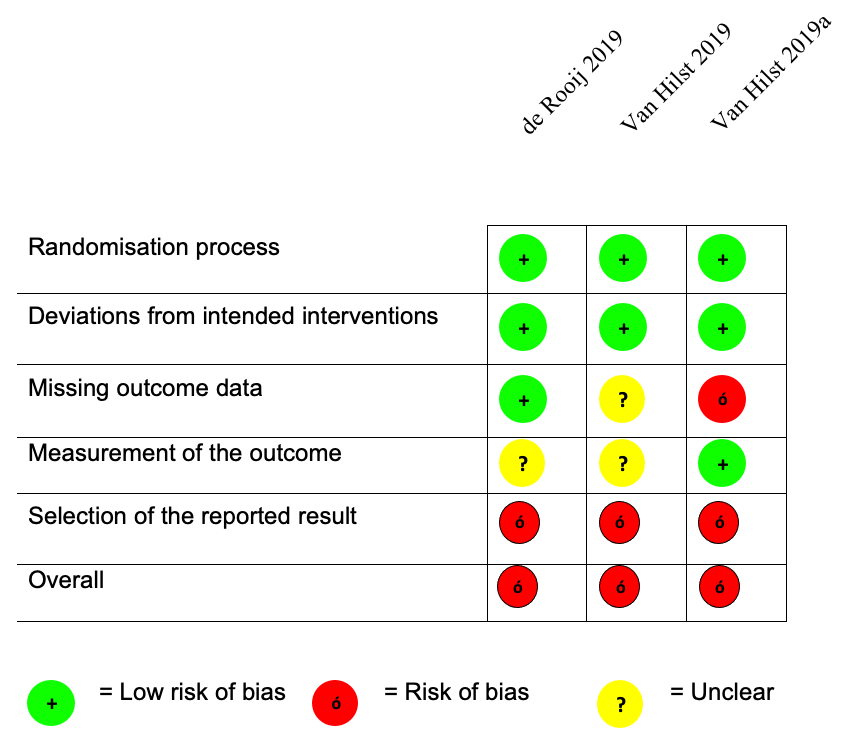
**

**Figure S3: Index Hospitalisation Lap Only Subgroup Funnel Plot**

**Table S1: Summary of risk-of-bias domains for all included studies**

| **QOL studies** |  |  |  |  |  |  |  |  |  |
| --- | --- | --- | --- | --- | --- | --- | --- | --- | --- |
| **Author & Year** |  | **Study design** | **Sample size** | **Bias due to confounding** | **Bias due to selection of participants** | **Bias in the intervention** | **Bias in measurement of outcome** | **Bias due to missing data** | **Bias in selection of reported findings** |
| Braga 2015 | Risk rating (high/medium/low/unclear) | Case-matched study | Low | Low | Unclear | Unclear | Medium | Unclear | Low |
| de Rooij 2019 | Risk rating (high/medium/low/unclear) | RCT-based | Low | Low | High | Unclear | Low | Unclear | Low |
| Langan 2014 | Risk rating (high/medium/low/unclear) | Case-matched study | Medium | Unclear | High | Unclear | Low | Medium | Low |
| Ricci 2015 | Risk rating (high/medium/low/unclear) | retrospective cohort | Low | Low | Low | Unclear | Low | Medium | Low |
| Torphy 2019 | Risk rating (high/medium/low/unclear) | retrospective cohort | Low | Low | Unclear | Unclear | Low | Medium | Low |
| van Hilst 2019a | Risk rating (high/medium/low/unclear) | RCT | Low | Unclear | Unclear | Unclear | Low | High | Low |
| van Hilst 2019b | Risk rating (high/medium/low/unclear) | RCT | Low | Low | Unclear | Unclear | Low | Unclear | Low |
| Andrian 2012 | Risk rating (high/medium/low/unclear) | retrospective cohort | Low | Low | Medium | High | Unclear | Unclear | Medium |
| Baker 2015 | Risk rating (high/medium/low/unclear) | retrospective cohort | Low | Medium | Medium | Unclear | Unclear | Unclear | Low |
| Braga 2015 | Risk rating (high/medium/low/unclear) | case-matched study | Low | Low | Unclear | High | Unclear | Low | Low |
| de Rooij 2019 | Risk rating (high/medium/low/unclear) | RCT | Low | Low | High | High | Unclear | High | Low |
| Eguia 2019a | Risk rating (high/medium/low/unclear) | retrospective cohort | Low | High | High | Unclear | Unclear | Low | Low |
| Eguia 2019b | Risk rating (high/medium/low/unclear) | retrospective cohort | Low | High | High | Unclear | Unclear | Low | Low |
| Eom 2008 | Risk rating (high/medium/low/unclear) | case-control (2:1 matching) | Low | High | High | High | Unclear | High | Low |
| Fisher 2019 | Risk rating (high/medium/low/unclear) | retrospective cohort | Low | Unclear | Unclear | Unclear | Unclear | Low | Low |
| Gerber 2017 | Risk rating (high/medium/low/unclear) | retrospective cohort | Low | High | High | Low | Unclear | High | High |
| Kowalsky 2019 | Risk rating (high/medium/low/unclear) | retrospective cohort | Low | Unclear | Medium | Medium | Unclear | Unclear | Low |
| Liang 2015 | Risk rating (high/medium/low/unclear) | retrospective cohort | Medium | Low | Medium | Unclear | Unclear | Low | Low |
| Limongelli 2012 | Risk rating (high/medium/low/unclear) | retrospective cohort | Medium | Medium | Unclear | Low | Unclear | Unclear | Low |
| Mesleh 2013 | Risk rating (high/medium/low/unclear) | retrospective cohort | Low | Medium | Low | Unclear | Unclear | Low | Medium |
| Ricci 2015 | Risk rating (high/medium/low/unclear) | retrospective cohort | Low | Low | Unclear | Unclear | Unclear | Low | Low |
| Rodriguez 2018 | Risk rating (high/medium/low/unclear) | retrospective cohort | Low | Medium | Low | Unclear | Unclear | Low | Low |
| Rutz 2014 | Risk rating (high/medium/low/unclear) | retrospective cohort | Low | Low | Unclear | Unclear | Unclear | Low | Low |
| Tran 2016 | Risk rating (high/medium/low/unclear) | retrospective cohort | Low | Medium | High | Unclear | Unclear | High | Low |
| van Hilst 2019a | Risk rating (high/medium/low/unclear) | RCT | Low | Unclear | High | Unclear | Unclear | High | Low |
| van Hilst 2019b | Risk rating (high/medium/low/unclear) | RCT | Low | Low | High | Unclear | High | High | Low |
| Waters 2010 | Risk rating (high/medium/low/unclear) | retrospective cohort | Low | High | High | Unclear | High | High | Low |
| Xourafas 2015 | Risk rating (high/medium/low/unclear) | retrospective cohort | Low | High | High | High | Unclear | High | Low |
| Xourafas 2019 | Risk rating (high/medium/low/unclear) | retrospective cohort | Low | Medium | High | Unclear | Unclear | Low | Low |

**Table S2: Summary of costs of surgical equipment narratively reported**

| **Study author & year** | **Unit** | **Lap cost** | **Rob cost** | **Open cost** | **Difference in cost** | **P-value** |
| --- | --- | --- | --- | --- | --- | --- |
| **Lap vs Rob vs Open** | | | | | | |
| Rodriguez 2018^(118^**^)^** | Median (range) | 48  (48–48) | 2871 (2507–3724) | 35  (35–35) | - | **0.0001** |
| **Lap+Rob vs Open** | | | | | | |
| Xourafas 2019^(127^**^)^** | Ratio of median | - | | - | **1.65** | **<0.0001** |
| **Lap-only vs Open** | | | | | | |
| Braga 2015^(107^**^)^** | Mean | - | - | - | **1113** | **-** |
| Xourafas 2015^(126^**^)^** | Ratio of median | - | - | - | **1.93** | **<0.001** |

Lap, Laparoscopy; Rob, Robotic

Price converted to USD price-year 2020, except data presented as ratio of median

**Table S3: Summary of costs of index hospitalisation narratively reported**

| **Study author & year** | **Unit** | **Lap cost** | **Rob cost** | **Open cost** | **Difference in cost** | **P-value** |
| --- | --- | --- | --- | --- | --- | --- |
| **Lap vs Rob vs Open** | | | | | | |
| Rodriguez 2018^(118^**^)^** | Median (range) | 29551 (14610–131838) | 28309 (18419–48143) | 41263 (13123–158583) | - | **0.02** |
| **Lap+Rob vs Open** | | | | | | |
| Xourafas 2019^(127^**^)^** | Ratio of median | - | | - | **-0.11** | **0.0150** |
|  |  | - | | - | **-0.12*** | **0.0048** |
| **Lap-only vs Open** | | | | | | |
| Braga 2015^(107^**^)^** | Mean | - | - | - | **228** | **-** |
| Xourafas 2015^(126^**^)^** | Ratio of median | - | - | - | **0.12** | 0.091 |
| **Rob-only vs Open** | | | | | | |
| Baker 2015^(106^**^)^** | Median (range) | - | 159580  (127965–271916) | 153567 (95675–654669) | 1442235 | >0.05 |

*Lap, Laparoscopy; Rob, Robotic.*

*Price converted to USD price-year 2020.*

**Amortized cost for robotic system maintenance*

**Table S4: Characteristics of included studies assessing health-related quality of life**

| **Study** | **HRQoL endpoint** | **Intervention (n)** | **Conversion rate to open** | **Intension to treat** | **Follow up retention** | **QoL Instrument** | **Timing of instrument** |
| --- | --- | --- | --- | --- | --- | --- | --- |
| Braga 2015 | 2∘ | LDP (100) | 23.00% | Yes | 1 & 3 m: 100% | SF-8 | 1 and 3 m |
|  |  | ODP (100) |  |  |  |  |  |
| de Rooij 2019 | 2∘ | MIDP (47; 42 LDP, 5 RDP) | 8.00% | Yes | NR | EQ-5D-3L | 1, 3, 5, 14, 30, 90 days |
|  |  | ODP (55) |  |  |  | EORTC-QLQ-C30 | 14, 30, 90 days |
| Langan 2014 | 1∘ | LPD (28) | 0% | No converted cases included | 1-6m: Lap 32%, Open 44% | SF-36 | Multiple time points between 2-20 months |
|  |  | OPD (25) |  |  | 6-12m: Lap 32%; Open 28% | Karnofsky scores |  |
|  |  |  |  |  | 12-24m: Lap 36%; Open 28% |  |  |
| Ricci 2015 | 1∘ | LDP (41) | 12.20% | Yes | 1 y: 100% | EORTC-QLQ-C30 EQ-5D | 1 y |
|  |  | ODP (40) |  |  |  |  |  |
| Torphy 2019 | 1∘ | Lap (63, 37 LPD, 26 LDP)  Open (96, 77 OPD, 19 ODP) | 14.30% | Yes | 0d: Lap 98%, Open 96% | FACT-Hep | Baseline, 14, 30, 90, 180 days |
|  |  |  |  |  | 2w: Lap 92%, Open 73%. |  |  |
|  |  |  |  |  | 1m: Lap 89%, Open 74% |  |  |
|  |  |  |  |  | 3m: Lap 81%, Open 66% |  |  |
|  |  |  |  |  | 6m: Lap 73%, Open 67% |  |  |
| van Hilst 2019 | 1∘ | LPD (50) | 20% | Yes | NR | EQ-5D-3L | 2, 4, 6 days; 2, 4, 12 weeks |
|  |  |  |  |  |  | EQ-5D-3L VAS |  |
|  |  | OPD (49) |  |  |  | EORTC-QLQ-C30 | 2, 4, 12 weeks |
| van Hilst 2019a | 2∘ | MIDP (48; 42 LDP, 5 RDP)  ODP (56) | 2.10% | Yes |  | EQ-5D-3L VAS |  |
|  |  |  |  |  |  | EORTC-QLQ-C30 | 2, 4, 12 weeks |
|  |  |  |  |  | 1y: 60.6% | EORTC-QLQ-C30 | Baseline, 1, 3, 5, 14, 30, 90, 365 days |
|  |  |  |  |  |  | EQ-5D & 5L |  |

*HRQoL, Health-related quality of life; LDP, Laparoscopic distal pancreatectomy; LPD, Laparoscopic pancreaticoduodenectomy, MIDP, Minimally invasive distal pancreatectomy; ODP, Open distal pancreatectomy; OPD, Open pancreaticoduodenectomy; RDP, Robotic distal pancreatectomy; d, Day; Lap, Laparoscopic; m, Month; MCS, Mental Composite Score; MIDP, Minimally invasive distal pancreatectomy; NR, Not reported; PCS, Physical Composite Score; w, Week; y, Year.*

**Table S5: Summary of global HRQoL studies**

| **Questionn-aire** | **Author & year** | **Mean diff. (P-value)** | |
| --- | --- | --- | --- |
|  |  | **≤1 month** | **>1 month** |
| **SF-8** | Braga 2015  ^(128^**^)^** | 0.4  (**P=0.03**) | - |
| **EORTC-QLQ-C30** | de Rooij 2019^(129^**^)^** | 9.269  (**P=0.049**) | 0.948  (P=0.81) |
|  | van Hilst 2019b^(123^**^)^** | - | –2.69  (P=0.33) |
|  | Ricci 2015^(117^**^)^** | - | 6.3 |
|  | van Hilst 2019a^(130^**^)^** | - | 0⋅39  (P=0.905) |
| **EQ-5D** | de Rooij 2019^(129^**^)^** | 0.030  (P=0.59) | 0.037  (P=0.47) |
|  | van Hilst 2019b^(123^**^)^** | - | 0·01  (P=0.76) |
| **EQ-5D VAS** | de Rooij 2019^(129^**^)^** | 8.71  (**P=0.04**) | 3.15  (P=0.38) |
|  | van Hilst 2019b^(123^**^)^** | - | –2·48  (P=0.22) |
| **FACT-Hep** | Torphy 2019^(131^**^)^** | -11.44*  (P>0.05) | 1.02**  (P>0.05) |

*SF-8, Short Form Health Survey 8-Item; EORTC-QLQ-C30, European Organisation for Research and Treatment of Cancer Quality of Life Questionnaire Core 30; EQ-5D, EuroQoL 5-Dimension Questionnaire; VAS, Visual Analogue Scale; FACT-Hep, Functional Assessment of Cancer Therapy-Hepatobiliary questionnaire; Mean diff, Mean difference scores between minimally invasive and open.*

**up to 2 weeks postoperative. **from 2 weeks postoperative to*

*If there were multiple values from the same study in the same time period of <1 month and > 1 month, results of the latter timepoint were presented in the table.*

**Table S6: Summary of physical functioning HRQoL domain**

| **Questionnaire/ Score** | | **Author & year** | **Mean diff. (P-value)** | |
| --- | --- | --- | --- | --- |
|  |  |  | **≤1 month** | **>1 month** |
| **SF-8** | Physical functioning | Braga 2015^(128^**^)^** | 0.4  (P>0.05) | NR  (P>0.05) |
|  | Role physical |  | 0.7  (P>0.05) | NR  (P>0.05) |
| **SF-36** | PCS | Langan 2014^(113^**^)^** | - | 3.9  (P=0.07) |
| **Karnofsky score** | | Langan 2014^(113^**^)^** | - | 6.7%  (P=0.59) |
| **EORTC-QLQ-C30** | Physical functioning | Ricci 2015^(117^**^)^** | - | 15.4  **(P=0.046)** |
|  |  | van Hilst 2019b^(123^**^)^** | - | –4.30 (P=0.15) |
|  |  | de Rooij 2019^(129^**^)^** | 6.05 | 1.98 |
|  | Role physical | Ricci 2015^(117^**^)^** | - | 19.3  (**P=0.035**) |
|  |  | de Rooij 2019^(129^**^)^** | 8.3 | -0.911 |
| **FACT-Hep** | Physical | Torphy 2019^(131^**^)^** | -1.99* (P>0.05) | 0.07 **  (P>0.05) |
|  | Functional |  | -1.92*  (P>0.05) | 0.07**  (P>0.05) |

*SF-8, Short Form Health Survey 8-Item; SF-36, SF 36-item; EORTC-QLQ-C30, European Organisation for Research and Treatment of Cancer Quality of Life Questionnaire Core 30; FACT-Hep, Functional Assessment of Cancer Therapy-Hepatobiliary questionnaire; Mean diff, Mean difference scores between minimally invasive and open.*

**up to 2 weeks postoperative. **from 2 weeks postoperative to*

*If there were multiple values from the same study in the same time period of <1 month and > 1 month, results of the latter timepoint were presented in the table.*

**Table S7: Summary of physical pain and disease-specific symptoms domain of HRQoL**

| **Questionnaire/ Score** | | **Author & year** | **Mean diff. (P-value)** | |
| --- | --- | --- | --- | --- |
|  |  |  | **≤1 month** | **>1 month** |
| **Pain** | | | | |
| **SF-8** | Bodily pain | Braga 2015^(128^**^)^** | 0.6  (P>0.05) | NR  (P>0.05) |
| **EORTC-QLQ-C30** | Bodily pain | Ricci 2015^(117^**^)^** | - | -6.4  (P>0.05) |
|  |  | van Hilst 2019b^(123^**^)^** | - | 5.89  (**P=0.07**) |
|  |  | de Rooij 2019^(129^**^)^** | -10.83 | -1.95 |
| **EORTC QLQ-PAN26** | Pancreatic pain | van Hilst 2019a^(130^**^)^** | - | 3.6  (P-0.216) |
|  | Hepatic pain | van Hilst 2019a^(130^**^)^** | - | -2.3 (P=0.324) |
| **Other symptoms** | | | | |
| **EORTC-QLQ-C30** | Fatigue | Ricci 2015^(117^**^)^** | - | -8.3  (P>0.05) |
|  |  | de Rooij 2019^(129^**^)^** | -8.18 | 0.93 |
|  | Nausea/vomiting | Ricci 2015^(117^**^)^** | - | 2.2  (P>0.05) |
|  |  | de Rooij 2019^(129^**^)^** | -0.97 | -0.25 |
|  | Dyspnea | Ricci 2015^(117^**^)^** | - | -10.0  (P>0.05) |
|  |  | de Rooij 2019^(129^**^)^** | -1.09 | 1.82 |
|  | Appetite loss | Ricci 2015^(117^**^)^** | - | 1.7  (P>0.05) |
|  |  | de Rooij 2019^(129^**^)^** | -0.91 | 3.38 |
|  | Sleep disturbance | Ricci 2015^(117^**^)^** | - | -14.0 |
|  |  | de Rooij 2019^(129^**^)^** | 0.26 | 9.7 |
|  | Constipation | Ricci 2015^(117^**^)^** | - | 4.2  (P>0.05) |
|  |  | de Rooij 2019^(129^**^)^** | -9.41 | -5.9 |
|  | diarrhoea | Ricci 2015^(117^**^)^** | - | 5.9  (P=0.032) |
|  |  | de Rooij 2019^(129^**^)^** | -1.53 | -2.97 |
| **EORTC QLQ-PAN26** | Digestive | van Hilst 2019a^(130^**^)^** | - | 5.6 (P=0.153) |
|  | Altered bowel habit | van Hilst 2019a^(130^**^)^** | - | 4.5  (P=0.258) |
| **FACT-Hep** | Hepatic | Torphy 2019^(131^**^)^** | - 5.54* (P>0.05) | 0.38** (P>0.05) |

*SF-8, Short Form Health Survey 8-Item; EORTC-QLQ-C30, European Organisation for Research and Treatment of Cancer Quality of Life Questionnaire Core 30; QLQ-PAN26, Pancreatic Cancer Module; FACT-Hep, Functional Assessment of Cancer Therapy-Hepatobiliary questionnaire; Mean diff, Mean difference scores between minimally invasive and open.*

** up to 2 weeks postoperative. ** from 2 weeks postoperative to*

**Table S8: Summary of psychological wellbeing domain of HRQoL**

| **Questionnaire** | **Author & year** | **Mean diff. (P-value)** | |
| --- | --- | --- | --- |
|  |  | **≤1 month** | **>1 month** |
| **Mental health/ cognitive functioning** | | | |
| **SF-8** | Braga 2015^(128^**^)^** | 0.4 | NR |
|  |  | (P>0.05) | (P>0.05) |
| **SF-36** | Langan 2014^(113^**^)^** | 8.5 | -0.4 |
|  |  | (P=0.17) | (P=0.94) |
| **EORTC QLQ-C30** | Ricci 2015^(117^**^)^** | - | 9.9 |
|  |  |  | (P>0.05) |
|  | de Rooij 2019^(129^**^)^** | -4.7 | -0.28 |
| **Emotion** | | | |
| **SF-8** | Braga 2015^(128^**^)^** | 0.4 | NR |
|  |  | (P>0.05) | (P>0.05) |
| **FACT-Hep** | Torphy 2019^(131^**^)^** | -3.05* | 0.42** |
|  |  | (**P<0.05**) | (**P<0.01**) |
| **EORTC QLQ-C30** | Ricci 2015^(117^**^)^** | - | 8.6 |
|  |  |  | (P>0.05) |
|  | de Rooij 2019^(129^**^)^** | 0.37 | -2.15 |

*SF-8, Short Form Health Survey 8-Item; SF-36, SF 36-item; European Organisation for Research and Treatment of Cancer Quality of Life Questionnaire Core 30; FACT-Hep, Functional Assessment of Cancer Therapy-Hepatobiliary questionnaire; Mean diff, Mean difference scores between minimally invasive and open.*

** up to 2 weeks postoperative. ** from 2 weeks postoperative to*

*If there were multiple values from the same study in the same time period of <1 month and > 1 month, results of the latter timepoint were tabulated.*

**Table S9: Summary of social functioning domain of HRQoL**

|  | **Author & year** | **Mean diff. (P-value)** | |
| --- | --- | --- | --- |
| **Questionnaire** |  | **≤1 month** | **>1 month** |
| **SF-8** | Braga 2015^(128^**^)^** | 0.6(P>0.05) | NR (P>0.05) |
| **EORTC QLQ-C30** | Ricci 2015^(117^**^)^** | - | 4.0 (P>0.05) |
|  | de Rooij 2019^(129^**^)^** | 10.22 | 2.0 |
| **FACT-Hep** | Torphy 2019^(131^**^)^** | 0.82* (P>0.05) | 0.08** (P>0.05) |

*SF-8, Short Form Health Survey 8-Item; European Organisation for Research and Treatment of Cancer Quality of Life Questionnaire Core 30; FACT-Hep, Functional Assessment of Cancer Therapy-Hepatobiliary questionnaire; Mean diff, Mean difference scores between minimally invasive and open.*

**Table S10: Summary of self-perceptions on wound cosmesis and pain domain of HRQoL**

| **Score** | **Author & year** | **1 year** | |
| --- | --- | --- | --- |
|  |  | **Mean diff.** | **P-value** |
| EORTC QLQ-PAN26  Body image | van Hilst 2019a^(130^**^)^** | 1.2 | P=0.607 |
| cosmetic satisfaction score | van Hilst 2019a^(130^**^)^** | 2* | P=0.506 |
| No. people visiting from scar |  | 1 | P=0.728 |
| Chronic scar pain:  at rest |  | 0* | P=0⋅433 |
| during movement |  | 0* | P=0.993 |
| during coughing/lifting heavy object |  | 0* | P=0.259 |
| effect on daily activities |  | 0* | P=0.426 |

*EORTC-QLQ-PAN26, European Organisation for Research and Treatment of Cancer Quality of Life Questionnaire Pancreatic Cancer Module; Mean diff, Mean difference scores between minimally invasive and open.*

**Median*
